# Dialectical materialism and teaching by words and deeds applied to the education of clinical medical students

**DOI:** 10.3389/fmed.2023.1037101

**Published:** 2023-07-24

**Authors:** Meng Li, Ali Sorayyaei Azar

**Affiliations:** ^1^Graduate School of Management, Management and Science University, Shah Alam, Selangor, Malaysia; ^2^Academy of Marxism, Henan Open University, Zhengzhou, Henan, China; ^3^Department of Education, School of Education and Social Sciences, Management and Science University, Shah Alam, Selangor, Malaysia

**Keywords:** empathy, materialistic dialectics, recall bias, training clinicians, training model

The application of materialist dialectics in the field of medical education is increasingly extensive. An excellent medical training model includes requirements for both training clinicians and medical students. Training clinicians requires teachers to lead by example and through verbal instruction, while medical students require strong interpersonal skills and empathy. A training clinician's recollection of a medical student may determine their overall evaluation of the student.

Materialist dialectics is increasingly widely used in the field of medical education. In particular, the study by Steinmair et al. (1) proposed that practitioners in the healthcare field not only need to continuously improve their professional quality but also need to maintain their ability to engage in lifelong learning. In this study, a total of 33 training clinicians were invited to mentor medical students and evaluate their behavioral performance, including their professional knowledge, soft skills (i.e., interpersonal skills), system skills, and environmental background factors. Through an analysis of the assessment results, the authors pointed out that only when theoretical knowledge is combined with professional practice can doctors better communicate with patients and obtain a more detailed medical history from them. In addition, they found that empathy may be a distinct skill independent of professionalism and that the level of empathy may vary by physician's gender. Although the work of the author and his team has been quite sufficient, and the conclusions drawn are supported by a considerable amount of literature, after our discussion and study, we determine that there are still some views and proposals in this article that deserve further exploration.

A good medical training model not only requires medical students to have a positive learning character but also includes certain requirements for training clinicians. We have supplemented the author's study, and our supplementary content is based on the research results of Hoffert. Hoffert et al. (2), whose study pointed out that training a clinician requires a complex curriculum and instruction and that the quality of teaching greatly affects the training results. Although training clinicians involved in mentoring medical students have spent time working in the field and have achieved some career results, they often do not have formal training in effective teaching practices. Thus, Hoffert MM designed an original teacher development program that is closer to clinical practice than basic educational theory. In it, five key aspects of effective teaching in relation to clinical settings are distilled: a culturally humble and safe learning environment, instructional practices that engage learners, coaching and assessment strategies, receiving and providing feedback, and coaching and mentoring. A central feature of the program is that training clinicians actively implement these five elements in their behavior and integrate them into their daily lives, whereby they emphasize the importance of precept and example.

On the one hand, the relationship between training clinicians and clinical medical students may directly affect the training clinicians' impressions of them, which, in turn, affects the training clinicians' evaluation results of the visit overall (3–5). On the other hand, the study results of this experiment are derived from the recall of training clinicians, and the author does not specify the time period of this recall in the text. Here, we need to point out that the longer the recall period is, the lower the authenticity and reliability of the results become, so we recommend that the authors supplement the recall period for training clinicians. In statistics, this issue is referred to as recall bias (6). Recall bias is mainly derived from the distortion of the object's memory content or incomplete recall. Ultimately, the accuracy or completeness of the experimental data will be reduced by this, resulting in large systematic errors.

Speculation is an important part of conducting research (7). From a static point of view, communication is a universal social relationship formed between people. Materialist dialectics emphasizes that connections exist widely, and all things that are connected must necessarily interact with each other. The content of the communication is determined by each individual's thinking, and the way of communication is closely related to the individual's behavior. A study by Park and Young (8) reports that the activity of the right temporoparietal junction (RTPJ) underlies the involvement of moral renewal in the social brain. By investigating whether RTPJ activity is related to maintaining close relationships, they found that RTPJ activity was lower when friends acted negatively toward subjects and relatively high when strangers acted negatively toward them. This suggests that the selective ignorance of people's negative behaviors in the context of intimate relationships is related to maintaining intimacy, and this selective ignorance may also have potential social benefits.

Although the author notes that the mention of “empathy” may be related to the gender of the guiding doctor and points out that women may pay greater attention to the literacy of “empathy” [more GPs and psychiatrists mentioned attitude and empathy in the context of 'ideal history taking,' with a higher proportion of women (1)], the author did not use the original data to compare this difference between groups. Due to our lack of raw data, the statistical analysis suggested above could not be conducted. Therefore, we suggest that the authors use gender as the grouping basis, mark cases that mention “empathy” as positive and those that do not mention “empathy” as negative, perform a group *t*-test, and supplement the *t*-value and *p*-value. Statements backed up by data and statistics are generally more convincing.

## Conclusion

An excellent medical training model includes requirements for both training clinicians and medical students. Training clinicians are required to teach by personal example, as well as through verbal instruction, and medical students need to have strong interpersonal skills and empathy. A training clinician's recall impression of a medical student may determine their overall evaluation of the student due to recall bias, and thus, there may be a deviation between the result and the true value. Making gender-related conclusions requires corresponding statistical analysis results.

## Author contributions

ML: conceptualization, writing of the original draft, and formal analysis. AS: reviewing and editing. All authors participated in the drafting of the manuscript, and all have read, contributed to, and approved the final version of the manuscript.
